# Special Issue “Editorial Board Members’ Collection Series: Cancer Metastasis”

**DOI:** 10.3390/ijms27093910

**Published:** 2026-04-28

**Authors:** Carlos Cabañas

**Affiliations:** Tissue and Organ Homeostasis Program, Cell-Cell Communication Unit, Centro de Biología Molecular Severo Ochoa, Consejo Superior de Investigaciones Científicas—Universidad Autónoma de Madrid (CSIC—UAM), Nicolás Cabrera 1, 28049 Madrid, Spain; ccabanas@cbm.csic.es

Cancer metastasis is the process by which neoplastic cells from a primary tumor spread to distant sites, where cancer cells establish and form new tumors, termed secondary tumors or metastases. Neoplastic cells acquire metastatic capacity through the metastatic cascade, a multi-step process generally comprising proliferation and local invasion at the primary tumor, access to a transport route (hematogenous, lymphatic, transcoelomic), and extravasation and colonization of distant organs [1,2,3]. Once in distant organs, surviving cancer cells adapt to the new microenvironment, induce angiogenesis and expand into detectable secondary tumors. Metastasis is a hallmark of cancer and is the primary cause of cancer-related morbidity and mortality, because secondary tumors disrupt organ function and are harder to eradicate. Metastasis outcomes vary widely by cancer type, the number and location of metastases, and available therapies. Importantly, current research on cancer metastasis focuses on understanding the molecular steps of the metastatic cascade, identifying biomarkers that predict metastatic risk, and developing therapies that target specific stages of the metastatic cascade. In recent years, it has become increasingly evident that cancer metastasis is not merely the consequence of genetic alterations within tumor cells, and that dynamic interactions among cellular plasticity, stromal ecosystems, and organ-specific molecular gateways also play a major role in the metastatic process [1,2,3]. Therefore, although recent advances in precision medicine and immunotherapy have significantly improved outcomes for some metastatic cancers, major challenges still remain in preventing and eradicating established metastases [4,5].

This Special Issue on cancer metastasis in the *International Journal of Molecular Sciences* brings together seven contributions that collectively advance our understanding of the molecular determinants governing cancer progression, metastatic dissemination, and therapeutic vulnerability. These seven articles span diverse tumor types and methodological approaches, yet converge on a shared theme: cancer is a dynamic, adaptive system shaped by the interplay between cellular plasticity, microenvironmental cues, and molecular signaling networks. The three research studies in this Special Issue reveal (a) how epithelial–mesenchymal transition (EMT) modulates bioelectric susceptibility in lung cancer cells; (b) how stromal activation markers such as fibroblast activation protein-alpha predict recurrence and drug resistance in renal cell carcinoma; and (c) how preoperative molecular profiling can refine risk assessment in papillary thyroid microcarcinoma. Additionally, the four review articles in this Special Issue contribute to deepening our understanding of (d) melanoma brain metastasis biology; (e) leader-cell biology and dynamics in cancer invasion and progression; (f) the promise and limitations of emerging targeted therapies for EGFR exon 20 insertions in NSCLC; and (g) the molecular determinants that dictate tumor-derived extracellular vesicle (TEV) tropism.

Mitsui et al. investigate how epithelial–mesenchymal transition (EMT) influences the cellular response of human A549 lung carcinoma cells to nanosecond pulsed electric fields (nsPEFs), a physical treatment increasingly explored for cancer therapy. nsPEFs are ultrashort, high-intensity electrical pulses capable of generating membrane nanopores, disrupting ionic homeostasis, impairing mitochondrial function, and inducing cell death. The authors find that nsPEFs predominantly induced non-apoptotic cell death in EMT cells, highlighting a distinct vulnerability associated with EMT. To induce EMT, A549 cells were treated with TGF-β1 for 48 h, resulting in characteristic morphological and molecular changes: loss of epithelial markers (E-cadherin), increased mesenchymal markers (vimentin, fibronectin), and reorganization of the actin cytoskeleton. These changes were confirmed through fluorescence imaging and qRT-PCR. When exposed to 15 kV/cm nsPEFs, EMT-induced cells exhibited significantly reduced viability compared to non-EMT cells. This heightened susceptibility was specific to nsPEFs, as EMT-induced cells did not show increased sensitivity to UV irradiation and were even more resistant to paclitaxel. The authors attribute this specificity to EMT-associated membrane remodeling and demonstrate that nsPEFs generated substantially more nanopores in EMT cells, indicating increased membrane fragility. Further analysis revealed that nsPEFs induced non-apoptotic cell death in EMT-induced cells, as evidenced by the absence of caspase-3 and PARP-1 cleavage. Extracellular calcium enhanced nsPEF cytotoxicity in both EMT and non-EMT cells, supporting the hypothesis that nanopore-mediated Ca^2+^ influx contributes to cell death. In this study, the authors conclude that (i) EMT significantly increases susceptibility to nsPEFs in A549 cells, leading to greater loss of viability and enhanced nanopore formation; (ii) this increased vulnerability is specific to nsPEFs, not to general cellular stress; (iii) EMT-associated membrane and cytoskeletal remodeling likely underlies increased membrane fragility; (iv) nsPEFs induce non-apoptotic, Ca^2+^-dependent cell death in EMT cells. These findings suggest that nsPEFs may preferentially target EMT-like, invasive cancer cell populations, strengthening their potential as a novel therapeutic modality.

Riaza-Montes et al. have evaluated stromal fibroblast activation protein alpha (FAP) expression via immunohistochemistry in primary tumors from 137 patients with advanced clear cell renal cell carcinoma (ccRCC) and correlated FAP status with clinicopathological features, survival, and response to first-line tyrosine kinase inhibitors (TKIs). The cohort included both synchronous and metachronous metastatic cases treated with sunitinib, sorafenib, or pazopanib and was followed-up long-term (mean follow-up ≈ 67 months). FAP was scored qualitatively as present or absent in cancer-associated fibroblasts (CAFs) on tissue microarrays. The authors found that FAP positivity was significantly more frequent in high-grade tumors and in non-organ-confined (pT3–pT4) and stage III–IV cancers, whereas no consistent association with tumor diameter, IMDC (International Metastatic Renal Cell Carcinoma Database Consortium) risk, or ECOG (Eastern Cooperative Oncology Group) status was observed. Importantly, treatment response analysis showed that tumors achieving a complete response by RECIST (Response Evaluation Criteria in Solid Tumors) were much less likely to be FAP-positive. Survival analyses demonstrated that FAP positivity correlated with shorter disease-free survival (DFS) and reduced overall survival (OS) in univariate models; however, FAP did not remain an independent predictor in multivariate Cox models, where age, grade and pT stage (for DFS) and classical staging variables (for OS) prevailed. In silico analysis supported the clinical findings, showing higher FAP mRNA in tumors versus normal kidney and associations with worse survival and higher stages. In this study, the authors conclude that (i) FAP expression marks an aggressive stromal phenotype in advanced ccRCC and is associated with local invasion and higher histological grade; (ii) FAP positivity predicts poorer radiologic response to TKIs and correlates with shorter DFS and OS in univariate analyses; (iii) FAP is not an independent prognostic factor in multivariate models dominated by established pathological variables, but it likely reflects biologically relevant stromal processes that promote invasion and therapy resistance; and (iv) FAP IHC could help stratify patients and identify those who might benefit from stromal-targeted strategies or combination approaches.

The study by Lukyanov et al. evaluates whether molecular markers measured in preoperative fine-needle aspiration biopsy (FNAB) cytology can predict lymphogenic metastasis in papillary thyroid microcarcinoma (PTMC). Ninety-two patients with histologically confirmed PTMC were analyzed; thirty-two had regional cervical lymph node metastases. The authors quantified 33 molecular features (18 mRNAs, 13 microRNAs, BRAF V600 mutations, and mtDNA) from archival cytology specimens using RT-PCR and allele-specific assays. Statistical comparisons identified 11 markers significantly associated with metastatic status: upregulated HMGA2, TIMP1, FN1, and miR-146b; downregulated miR-7 and miR-148b; and reduced expression/activity of DIO1, TFF3, TPO, and SLC26A7. The BRAF V600E mutation was more frequent in metastatic cases (OR 4.6, *p* = 0.0016). Receiver operating characteristic analysis showed AUCs > 0.70 for miR-146b, miR-7, HMGA2, and miR-148b; optimal thresholds were defined by Youden indices. Diagnostic performance was characterized by high sensitivity (84.5–90.6%) but low specificity (~41–50%) for most molecular markers, yielding excellent negative predictive values (≈97–99%) but poor positive predictive values. The authors note that molecular testing on FNAB material could enhance existing risk-grading systems and be applied preoperatively. In this study, the authors conclude that (i) molecular profiling of routine FNAB cytology reliably detects expression differences linked to metastatic PTMC and is technically accessible; (ii) selected markers (miR-146b, miR-7, HMGA2, miR-148b, BRAF) identify most metastatic cases, but many false positives limit standalone clinical use (leading to the high sensitivity but low specificity of the analysis); (iii) these assays are best used to supplement imaging and clinical criteria when deciding active surveillance versus surgery, especially to rule out metastasis rather than to confirm it; and (iv) larger, prospective cohorts and combined multivariable models (molecular + imaging + clinical features) are needed to improve specificity and validate thresholds before routine adoption of these assays.

Melanoma is a highly aggressive and metastatic cancer that poses significant challenges due to its propensity to spread to distant organs, with brain metastasis representing a particularly devastating complication [6,7]. In their review article, O’Toole et al. synthesize the current preclinical and clinical evidence on the molecular, cellular, and microenvironmental mechanisms driving melanoma metastasis, emphasizing mechanisms of blood–brain barrier traversal, tumor–stroma co-option, and brain-specific genomic and transcriptional programs. The review emphasizes the tumor microenvironment (TME) of brain metastases, compromised blood–brain barrier (BBB), dynamic tumor-associated macrophage populations (microglia- and monocyte-derived macrophages), neutrophil and B-cell contributions, and stromal reprogramming that fosters immune suppression and ECM remodeling. Multi-omic and single-cell studies reveal brain-specific transcriptional programs and pre-metastatic subpopulations with neural adhesion signatures. The authors evaluate preclinical models—cell lines, PDX, organoids, 3D cultures, zebrafish, and genetically engineered mouse models—highlighting strengths and limitations for modeling BBB traversal, immune interactions, and therapeutic testing. They also summarize advances in therapeutic strategies to combat melanoma brain metastasis including targeted therapies with small molecules (BRAF/MEK inhibitors), immunotherapies, and combination approaches, noting improved intracranial responses but persistent resistance. The review further discusses emerging targets (PI3K/AKT, FAK), strategies to modulate the TME, and the need for agents that penetrate the CNS. Finally, the authors identify critical research gaps, including the need for brain-metastasis-specific clinical trials, AI-driven predictive models, and preventive strategies, to guide future efforts in improving outcomes for patients with melanoma brain metastasis.

Metastasis is dependent on collective invasion—a developmental process exploited by many epithelial cancers to establish secondary tumors and promote widespread disease [8,9]. The review article by Doran et al. highlights leader cells as key drivers of collective invasion in cancer metastasis. Leader cells (LCs) are a functionally distinct subpopulation of cells that direct migration, cellular contractility, and lead trailing or follower cells. LCs orchestrate collective invasion and contribute to metastasis across multiple epithelial cancers. LCs are morphologically polarized, form actin-rich protrusions (invadopodia/filopodia), engage ECM via β1 integrins and focal adhesions, and generate actomyosin-driven traction that pulls follower cells (FCs) during cohesive migration. Their emergence is regulated by ECM properties (stiffness, collagen architecture) and reciprocal LC-driven ECM remodeling (fibronectin secretion, LOXL3 deposition, protease delivery). LCs show metabolic specialization—preferential oxidative phosphorylation with mitochondrial trafficking to the leading edge—enabling the high ATP demand of invasion and dynamic leader–follower role switching tied to energy state and cell-cycle phase. A conserved marker of many LCs is keratin 14 (KRT14). KRT14+ cells are enriched at invasive fronts, required for collective invasion in multiple models, and correlate with metastatic potential. LCs commonly display hybrid EMT (epithelial–mesenchymal transition) states, co-expressing epithelial markers (e.g., KRT14, p63) with variable EMT transcriptional programs across cancer types, which underpins their phenotypic plasticity. This plasticity confers stem-like traits and chemoresistance—LCs display sphere-forming capacity, express stem markers (ALDH1A1, CD44v6, SOX2), and are enriched after chemotherapy, driving tumor repopulation. Importantly, LCs actively modulate the immune microenvironment by downregulating antigen-presentation pathways, reprogramming NK cells toward metastasis-supportive states, recruiting immunosuppressive myeloid cells (TAMs, PMN-MDSCs), and expressing immune checkpoints (PD-L1, CD200) that further suppress anti-tumor immunity. The authors note that while KRT14 is tightly associated with LC identity, its mechanistic role remains incompletely defined. The authors conclude that LCs are central drivers of collective metastasis and represent a therapy-resistant, plastic subpopulation that promotes invasion, immune evasion, and recurrence. However, key gaps in LC knowledge still remain, including the precise functional role of KRT14, the in vivo impact of disrupting mitochondrial trafficking on metastasis, and strategies to prevent LC-driven immune reprogramming. Targeting LC-specific vulnerabilities (ECM interactions, mitochondrial OXPHOS/mitochondrial trafficking, KRT14-associated pathways, or LC-mediated immunosuppression) may offer promising avenues for anti-metastatic therapies and could yield clinically actionable interventions.

Based on the latest available data, lung cancer remains the leading cause of cancer-related death globally, with roughly 1.8 million deaths annually, heavily dominated by NSCLC subtypes [10,11,12]. Non-small-cell lung cancer (NSCLC) cells frequently harbor mutations in the epidermal growth factor receptor (EGFR), with exon-20 insertions comprising 1–10% of these mutations. The review article by Seo et al. examines current diagnostic approaches and targeted therapies for EGFR exon-20 insertion mutations (ex20ins) in NSCLC. Ex20ins mutations are heterogeneous in sequence and structure, typically located in the loop following the αC helix of the EGFR kinase domain, and their structural changes sterically hinder binding of many first- and second-generation tyrosine kinase inhibitors (TKIs). The authors emphasize that next-generation sequencing (NGS)—including plasma NGS—is superior to conventional PCR for detecting the broad spectrum of ex20ins variants and for guiding therapy selection. The review summarizes clinical data for several agents developed to overcome ex20ins resistance: amivantamab (an EGFR/MET bispecific antibody) shows meaningful activity and is FDA-approved post-platinum therapy, with improved outcomes when combined with chemotherapy; mobocertinib (TKI) demonstrated activity and received accelerated approval but failed to show superiority in a first-line phase III trial; poziotinib (TKI) has mutation-location-dependent efficacy and notable toxicity; and zipalertinib and sunvozertinib are newer TKIs with promising response rates and manageable safety in early trials. The review also highlights ongoing clinical trials testing therapeutic combinations and first-line strategies. Despite recent therapeutic advances, key clinical challenges remain in targeted therapies for ex20ins in NSCLC, including the intrinsic heterogeneity of ex20ins variants, improvements in CNS penetration, and the emergence of resistance mutations. These challenges underscore the need for dynamic molecular monitoring, and future work must optimize mutation-specific drug selection, development of CNS-penetrant therapeutic agents, and combination strategies to improve durable outcomes for ex20ins in NSCLC.

Extracellular vesicles produced by tumor cells (TEVs) influence different stages of cancer development and spread, including tumorigenesis, cancer progression, and metastasis [13,14,15]. TEVs can trigger profound phenotypic and functional changes in target cells through three main general mechanisms: (i) docking of TEVs on target cells and triggering of intra-cellular signaling; (ii) fusion of TEVs and target cell membranes with release of TEV molecular cargo in the cytoplasm of the recipient cell; and (iii) uptake of TEVs by recipient cells. Through these processes, TEVs contribute to reprogramming the tumor microenvironment and promote metastasis at distant organs [13,14,15]. The review article contributed by Clares-Pedrero et al. synthesizes mechanistic and functional evidence that specific surface molecules on TEVs determine their cellular and organ tropism, thereby shaping metastatic patterns and immune modulation. Integrins on TEVs are highlighted as major determinants of organ-specific uptake, guiding TEVs to lung, liver, or brain niches and activating pro-metastatic signaling. TEV-associated fibronectin and heparan sulfate proteoglycans form part of a protein corona that further mediates adhesion via cellular receptors. The immunoglobulin family member ICAM-1 on TEVs engages leukocyte β2 integrins (LFA-1, Mac-1) to modulate antigen presentation, T cell priming, or immune suppression. The adhesion molecules ALCAM/CD166 and CD44 on TEVs promote their docking and functional transfer, which enhance invasion, mesothelial clearance, metabolic reprogramming, and chemoresistance in recipient cells. The metalloproteases ADAM10/ADAM17 may play a dual role, acting as adhesive ligands (disintegrin domain-binding integrins) and as sheddases that alter the balance between membrane and soluble forms of adhesion/signaling molecules on both TEVs and target cells. Finally, tetraspanin CD9 modulates these interactions by clustering receptors, inhibiting ADAM17 activity, or altering receptor conformation, thereby either promoting or restraining TEV docking and uptake depending on context. This review highlights that specific molecular determinants on the surface of TEVs (integrins, Ig-CAMs, CD44, ADAMs, tetraspanins) critically dictate their tropism and downstream pro-tumoral effects, and suggests that targeting these surface molecules or the TEV protein corona may offer therapeutic opportunities to block pre-metastatic niche formation, reverse immune suppression, or improve exosome-based delivery. Translational progress derived from the knowledge of these TEV surface molecules will require better in vivo validation, standardized EV characterization, and clinical strategies that consider TEV heterogeneity and dynamic protein coronas.

Together, the seven contributions within this Special Issue on cancer metastasis reflect the rapidly evolving landscape of molecular oncology and point toward new opportunities for therapeutic innovation in cancer metastasis. We truly hope that this Special Issue will serve as a valuable resource for researchers, clinicians, and translational scientists working to transform molecular insights into improved patient outcomes.
